# Single-Cell RNA Sequencing Reveals Molecular Differences Underlying Meiotic Competence of Brilliant Cresyl Blue (BCB) Selected Alpaca Oocytes

**DOI:** 10.3390/ani16193081

**Published:** 2026-09-30

**Authors:** Juana Quispe, Zeze Bravo, Luz Toribio-Alomia, Samuel Cusihuallpa-Cuchon, Gloria Levano, Jonathan LaMarre, Martha Valdivia

**Affiliations:** 1 Laboratory of Reproductive Physiology, Department of Zoology, Faculty of Biological Sciences, Universidad Nacional Mayor de San Marcos, Lima 15081, Peru; juanalady.quispe@unmsm.edu.pe (J.Q.); zbravog@unmsm.edu.pe (Z.B.); luzandrea.toribio@unmsm.edu.pe (L.T.-A.); gloria.levano1@unmsm.edu.pe (G.L.); 2 Faculty of Biological Sciences, Universidad Nacional de San Agustín de Arequipa, Arequipa 04001, Peru; scusihuallpa@unsa.edu.pe; 3 Department of Biomedical Sciences, Ontario Veterinary College, University of Guelph, Guelph, ON N1G 2W1, Canada; jlamarre@uoguelph.ca

**Keywords:** alpaca, oocyte meiotic competence, brilliant cresyl blue, single-cell RNA sequencing

## Abstract

Identifying oocytes with high meiotic competence remains a major challenge for improving in vitro embryo production in South American camelids. Although Brilliant Cresyl Blue (BCB) staining has been used to distinguish alpaca oocytes with different meiotic and developmental competence, the biological and molecular feature associated with their meiotic competence remain poorly understood. This study characterized the transcriptomic profiles of immature alpaca (*Vicugna pacos*) oocytes classified as BCB-positive (BCB+) or BCB-negative (BCB−) using single-cell RNA sequencing. BCB+ oocytes exhibited a significantly larger ooplasm diameter, a higher proportion of condensed chromatin configurations, and higher in vitro maturation rates than BCB− oocytes. Transcriptomic analysis identified 2978 differentially expressed genes and revealed a clear separation between groups (PC1 = 64.8%). Differentially expressed genes with higher transcript abundance in BCB+ oocytes were enriched in DNA metabolism, cell cycle, oocyte meiosis, and proteasome pathways, whereas those with higher transcript abundance in BCB− oocytes were enriched in gene expression regulation, RNA metabolism, chromatin remodeling, and intracellular signaling. Protein–protein interaction (PPI) network analysis identified distinct hub genes in each group, including *CDK1, CCNB2, MAD2L1*, and *AURKB* in BCB+ oocytes and *EP300*, *CREBBP*, *SMARCA4*, and *ARID1A* in BCB− oocytes. These results provide the first single-cell transcriptomic characterization of BCB-classified alpaca oocytes and establish the molecular basis underlying the biological differences identified by BCB staining.

## Simple Summary

Identifying oocytes with a greater ability to mature is important for improving embryo production in alpacas. Brilliant Cresyl Blue staining can help distinguish oocytes with different meiotic and developmental competence, but the biological differences underlying this classification are not fully understood. We compared immature alpaca oocytes classified using this staining method and analyzed gene activity in individual oocytes. Oocytes with greater meiotic competence were larger, showed differences in their nuclear organization, and were more likely to mature under laboratory conditions. They also showed increased activity of genes involved in cell division and oocyte maturation, whereas oocytes with lower meiotic competence showed greater activity of genes involved in the regulation of gene expression and cellular signaling. These findings provide a better understanding of the biological differences between alpaca oocytes identified by Brilliant Cresyl Blue staining and support its use as a tool for studying oocyte meiotic competence in this species.

## 1. Introduction

Oocyte developmental competence is defined as the ability of an oocyte to complete maturation, undergo fertilization, and support early embryonic development [1]. This competence is progressively acquired during oocyte growth and maturation through the accumulation of maternal messenger RNAs (mRNAs), proteins, organelles, and other essential factors required for post-fertilization developmental events [2,3,4]. A fundamental component of this process is the acquisition of meiotic competence, defined as the ability of the oocyte to resume and successfully complete nuclear maturation to the metaphase II (MII) stage.

Given their critical role in the success of assisted reproductive technologies, numerous morphological, metabolic, and molecular approaches have been developed to assess or predict oocyte meiotic and developmental competence [5,6,7]. Among these, Brilliant Cresyl Blue (BCB) staining has been used as a practical, non-invasive method for oocyte selection and as a visual marker of oocyte cytoplasmic maturation [8,9,10]. This method is based on the intracellular activity of glucose-6-phosphate dehydrogenase (G6PDH), a key rate-limiting enzyme in the pentose phosphate pathway. G6PDH activity contributes to the generation of NADPH and ribose-5-phosphate, which are involved in cellular biosynthesis and redox balance [11]. During the active growth phase of the oocyte, intracellular G6PDH activity is high, but it significantly decreases as oocytes complete their growth and acquire cytoplasmic competence [10,12]. Consequently, fully grown oocytes with lower G6PDH activity have a lower capacity to reduce BCB to a colorless form, retain a blue cytoplasmic coloration, and are classified as BCB-positive (BCB+), whereas growing oocytes with higher G6PDH activity reduce BCB to a colorless form and are classified as BCB-negative (BCB−) [10,13].

Numerous studies have demonstrated that BCB+ oocytes exhibit higher rates of nuclear maturation and in vitro blastocyst production across several species [14,15,16,17,18]. To explain these functional differences, previous studies have reported differences in maternal transcript abundance, mitochondrial number and function, and gene expression profiles between BCB+ and BCB− oocytes [19,20,21,22]. In alpacas, a species characterized by distinctive reproductive physiology and persistent limitations in in vitro embryo production [23,24], BCB staining has been shown to identify oocytes with different meiotic and developmental competence [25]; however, the molecular differences between BCB+ and BCB− oocytes associated with oocyte meiotic competence remain largely unknown.

Advances in gene expression profiling technologies have enabled a more comprehensive characterization of the molecular mechanisms associated with oocyte meiotic and developmental competence. Gene expression studies using microarrays, RT-qPCR, and next-generation sequencing (NGS) have identified numerous molecular markers and their associated biological functions [5,26]. In this context, single-cell RNA sequencing (scRNA-seq) has emerged as a high-resolution approach for characterizing the transcriptomic profiles of individual oocytes and assessing biological variability among them [27,28]. This approach provides new opportunities to investigate the molecular differences associated with the acquisition of meiotic competence.

In the present study, we used scRNA-seq to characterize the transcriptomic profiles of individual alpaca oocytes classified according to BCB staining and to identify the biological processes and molecular pathways associated with differences in meiotic competence between BCB+ and BCB− oocytes.

## 2. Materials and Methods

### 2.1. Ethical Considerations

All experimental procedures were conducted at the Laboratory of Reproductive Physiology, Faculty of Biological Sciences, Universidad Nacional Mayor de San Marcos (UNMSM), Lima, Peru. The study was approved by the Bioethics Committee of the Faculty of Biological Sciences, Universidad Nacional Mayor de San Marcos (approval code: No. 106-2024-CBE-FCB-UNMSM).

### 2.2. Chemicals

All reagents and chemicals were purchased from Sigma-Aldrich (St. Louis, MO, USA), unless otherwise stated.

### 2.3. Collection of Alpaca Oocytes

Alpaca ovaries were collected from adult females (4–6 years old) slaughtered at the municipal abattoir of Huancavelica, Peru (3680 m above sea level). Samples were transported in 0.9% physiological saline at 22 ± 1 °C in a sealed thermal container for 20–24 h to the Laboratory of Reproductive Physiology in Lima, Peru (161 m above sea level). Upon arrival, the ovaries were washed in sterile phosphate-buffered saline (PBS) prewarmed to 38.5 °C. Cumulus–oocyte complexes (COCs) were aspirated from 3–8 mm follicles using a 20-gauge needle attached to a 5-mL syringe containing 1 mL of prewarmed aspiration medium (HEPES-buffered TCM-199 (Gibco/BRL, Grand Island, NY, USA) supplemented with 10% fetal bovine serum (FBS), 0.3% polyvinyl alcohol (PVA), 0.015 mg/mL penicillin G sodium, and 0.025 mg/mL streptomycin). The follicular fluid was collected into 15-mL conical tubes and incubated at 38.5 °C for 15 min to allow COCs to settle. Subsequently, COCs were recovered from the bottom of the tube using a Pasteur pipette and transferred to a Petri dish for stereomicroscopic evaluation. The COCs were washed three times in 100-μL drops of aspiration medium. Finally, oocytes with a homogeneous cytoplasm and at least three layers of cumulus cells were selected for the different experiments.

### 2.4. Brilliant Cresyl Blue (BCB) Staining

**Figure 1:**
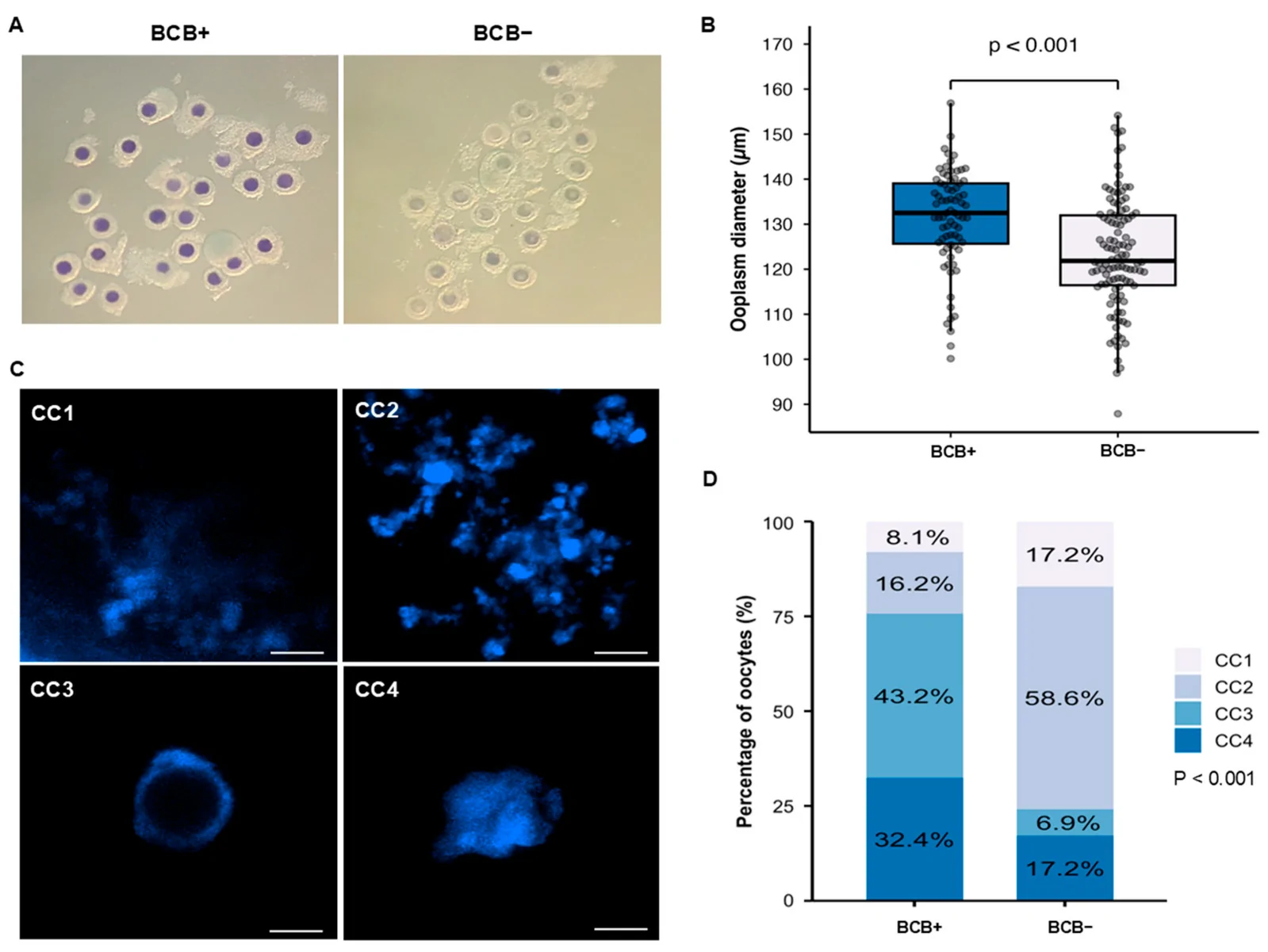
Morphological and nuclear characterization of oocytes classified by BCB staining. (**A**) Representative images of BCB+ (blue-stained cytoplasm) and BCB− (unstained cytoplasm) cumulus–oocyte complexes. (**B**) Ooplasm diameter of BCB+ and BCB− oocytes. Each dot represents an individual oocyte. (**C**) Representative images of chromatin configurations (CC1–CC4) according to the degree of chromatin condensation. Scale bar = 5 μm. (**D**) Distribution of chromatin configurations in BCB+ and BCB− oocytes.

Following morphological selection, immature COCs were washed in a 26 μM BCB solution prepared in Dulbecco’s phosphate-buffered saline supplemented with 0.3% PVA (DPBS-PVA). Subsequently, COCs were placed in groups of 5–10 in 50-μL drops of the same staining solution. The drops were covered with mineral oil, and the COCs were incubated for 90 min at 38.5 °C in a humidified atmosphere containing 5% CO_2_. After incubation, the COCs were washed three times in 100-μL drops of DPBS-PVA and classified by cytoplasmic staining as BCB-positive (BCB+, blue cytoplasm) or BCB-negative (BCB−, colorless cytoplasm) (Figure 1A). COCs with intermediate staining (slightly blue cytoplasmic coloration) were excluded from both groups.

### 2.5. In Vitro Maturation

COCs were allocated into four experimental groups: (1) Control (incubated in maturation medium without prior handling in DPBS-PVA), (2) BCB-Control (incubated in DPBS-PVA without BCB dye), (3) BCB+, and (4) BCB− (both incubated in DPBS-PVA containing 26 μM BCB). After collection or BCB staining, COCs were cultured in groups of 5–10 in 50-μL drops of TCM-199 with Earle’s salts (Gibco/BRL, Grand Island, NY, USA) supplemented with 2.2 g/L sodium bicarbonate, 10 IU/mL equine chorionic gonadotropin (eCG; Novormon ^®^, Syntex, Buenos Aires, Argentina), 0.1 IU/mL human chorionic gonadotropin (hCG), 0.07 IU/mL follicle-stimulating hormone (FSH; Folltropin^®^, Bioniche Animal Health, Belleville, ON, Canada), 10% fetal bovine serum, 0.2 mM sodium pyruvate, 1 μg/mL β-estradiol, 10 ng/mL epidermal growth factor (EGF), and 50 μg/mL gentamicin. The drops were covered with mineral oil and incubated for 32 h at 38.5 °C in a humidified atmosphere containing 5% CO_2_. At the end of the maturation period, cumulus cells were removed by gentle pipetting in a 0.1% hyaluronidase solution prepared in aspiration medium. Oocytes exhibiting the first polar body were considered mature and classified as being at the metaphase II (MII) stage.

### 2.6. Assessment of Ooplasm Diameter and Nuclear Configuration

Ooplasm diameter was assessed in 181 immature oocytes classified as BCB+ (*n* = 75) or BCB− (*n* = 106). Images of the COCs were acquired using a confocal microscope (LSM 900, Carl Zeiss, Oberkochen, Germany) equipped with a 10× objective. Ooplasm diameter was measured using Fiji (ImageJ v. 1.54p, NIH, Bethesda, MD, USA) as the average of two perpendicular measurements taken for each oocyte, excluding the zona pellucida.

To assess chromatin configuration, 89 oocytes classified as BCB+ (*n* = 52) or BCB− (*n* = 37) were processed. The oocytes were denuded in 0.1% hyaluronidase prepared in DPBS-PVA, fixed in 4% paraformaldehyde for 30 min, and permeabilized with 0.3% Triton X-100 for 30 min, with intermediate washes in PBS. The oocytes were then incubated in the dark with Hoechst 33342 (NucBlue™ Live ReadyProbes™, Thermo Fisher Scientific, Waltham, MA, USA) for 15 min, washed three times in PBS, and analyzed using the same microscope equipped with a 40× objective and an Airyscan detector (LSM900 with Airyscan 2, Carl Zeiss, Oberkochen, Germany). Images were processed using the Airyscan Processing algorithm in ZEN software v.3.10 (Carl Zeiss, Oberkochen, Germany), and nuclear configuration was determined from optical sections (Z-stacks) and three-dimensional reconstructions. For descriptive analysis, the observed chromatin configurations were grouped into four categories (CC1–CC4) based on the degree of chromatin condensation and spatial organization, adapted from previously described patterns of chromatin organization in mammalian GV oocytes [29,30]. CC1 corresponded to a diffuse chromatin distribution throughout the nucleus; CC2 was characterized by multiple condensed chromatin aggregates distributed throughout the nucleus; CC3 showed chromatin partially or completely organized around a central region compatible with the nucleolus; and CC4 was characterized by highly condensed chromatin forming a compact structure.

### 2.7. Preparation of Oocytes for scRNA-Seq

Following BCB classification, BCB+ oocytes showing intense blue cytoplasmic staining and BCB− oocytes showing complete absence of blue cytoplasmic staining were selected for scRNA-seq. Selected immature oocytes were denuded by gentle pipetting in DPBS-PVA containing 0.1% hyaluronidase. The oocytes were then washed three times in calcium- and magnesium-free DPBS supplemented with 1% bovine serum albumin (BSA). Subsequently, the zona pellucida was removed by exposure to 50-μL drops of acidic Tyrode’s solution for 30 s. Zona-free oocytes were washed four times in DPBS supplemented with 0.1% BSA. Finally, each oocyte was individually transferred in a final volume of 1 μL into a 0.2-mL tube containing 9.5 μL of lysis buffer provided in the SMART-Seq^®^ mRNA Kit (Takara, Shiga, Japan). The same volume of DPBS/0.1% BSA washing buffer was added to the lysis buffer and processed in parallel through cDNA synthesis and amplification as a negative control. The absence of amplified cDNA was confirmed by Qubit quantification (Qubit 2.0 Fluorometer, Thermo Fisher Scientific, Waltham, MA, USA) and Bioanalyzer analysis (Agilent 2100 Bioanalyzer, Agilent Technologies, Santa Clara, CA, USA).

### 2.8. cDNA Synthesis and Library Preparation

The complete lysates of individual BCB− (*n* = 4) and BCB+ (*n* = 4) oocytes were subjected to reverse transcription using the SMART-Seq^®^ mRNA Kit (Takara, Shiga, Japan) according to the manufacturer’s instructions. To minimize technical variation and potential batch effects, all oocyte samples were obtained from a single ovary collection and processed under identical experimental conditions, with BCB+ and BCB− samples interspersed throughout the workflow from cell lysis to cDNA amplification and purification. Briefly, first-strand cDNA was synthesized from the total RNA of each oocyte using an oligo(dT) primer. Subsequently, cDNA was amplified by PCR for 17 cycles. The amplified cDNA was purified using NucleoMag NGS Clean-up and Size Select magnetic beads (Macherey-Nagel, Düren, Germany). The quality and size distribution of the purified cDNA were assessed using an Agilent 2100 Bioanalyzer with the High Sensitivity DNA Kit (Agilent Technologies, Santa Clara, CA, USA). For library preparation, 1 ng of amplified cDNA per sample was used with the Nextera XT DNA Library Preparation Kit (Illumina Inc., San Diego, CA, USA) according to the manufacturer’s protocol. All individual libraries were prepared together in a single batch. The resulting libraries were evaluated using the Agilent 2100 Bioanalyzer, quantified with a Qubit fluorometer), normalized and pooled at an equimolar concentration of 4 nM. Paired-end sequencing (2 × 150 bp) was performed on the Illumina NovaSeq X Plus platform (Illumina Inc.) in a single sequencing run by Novogene Co., Ltd. (Sacramento, CA, USA).

### 2.9. RNA-Seq Data Processing and Quality Control

Paired-end FASTQ reads were processed using Trimmomatic (v0.39) to remove adapter sequences and low-quality bases [31]. Quality filtering was performed using a sliding window approach (window size = 4, Q ≥ 20), low-quality bases at read ends (Q < 3) were trimmed, and reads shorter than 50 bp were discarded. Filtered reads were aligned to the alpaca reference genome (VicPac4, NCBI RefSeq GCF_048564905.1) using STAR (v2.7.11b) [32]. Before read quantification, missing gene_id attributes in the reference annotation (*n* = 7402) were assigned using the corresponding transcript_id, which matched the gene identifier in these entries. Alignments were filtered using Samtools (v1.19.2) to remove unmapped reads, secondary alignments, and low-quality alignments. Gene-level read counts were generated using featureCounts (v2.1.1) in paired-end mode considering exonic regions [33]. Fragments per kilobase of transcript per million mapped reads (FPKM) values were calculated for descriptive analyses of gene abundance, with genes showing FPKM > 1 in at least one sample considered expressed [34]. Raw counts were used for subsequent PCA and differential gene expression analyses.

### 2.10. Principal Component Analysis (PCA)

For PCA, genes with a total count ≥ 10 across BCB+ and BCB− samples were retained. The raw count matrix was analyzed in R (v4.4.3) using Seurat (v5) [35]. Data were log-normalized using the LogNormalize method with a scale factor of 1 × 10^6^. The 2000 most variable genes were identified using the vst method, scaled, and subsequently used for PCA.

### 2.11. Differential Gene Expression Analysis

For differential gene expression analysis, genes with a total count ≥ 10 across all samples were retained. The analysis was performed in R using the DESeq2 package (v1.50.2) [36]. *p*-values were adjusted using the Benjamini–Hochberg method to control the false discovery rate (FDR). Genes with an adjusted *p*-value (padj) < 0.05 and an absolute log_2_ fold change (|log_2_ fold change|) ≥ 1 were considered differentially expressed genes (DEGs).

To assess the stability of log_2_ fold change (log_2_FC) estimates against variations in sample composition, a sensitivity analysis using bootstrap resampling was performed following Degen and Medo [37]. Oocyte samples within each experimental group (BCB+ and BCB−) were independently resampled with replacement, maintaining the original sample size (*n* = 4 per group), for 100 bootstrap iterations. For each iteration, differential expression analysis was repeated using DESeq2, and Spearman’s rank correlation coefficient (ρ) was calculated between the log_2_FC estimates obtained from the resampled dataset and those from the original dataset for genes with available estimates in both analyses.

### 2.12. Functional Enrichment Analysis (GO and KEGG)

Functional enrichment analysis was performed in R using the clusterProfiler package (v4.18.4) [38]. Due to the limited functional annotation available for *Vicugna pacos*, *Bos taurus* was used as the reference organism for Gene Ontology (GO) and Kyoto Encyclopedia of Genes and Genomes (KEGG) pathway analyses to improve annotation coverage. Alpaca gene symbols were mapped to *Bos taurus* Entrez IDs using the bitr function and the org.Bt.eg.db annotation package (v3.22.0) prior to enrichment analysis. Unmapped genes were categorized according to gene type, and the protein-coding fraction was further characterized by orthology-based functional annotation using eggNOG-mapper (v3 beta6) [39]. To assess the influence of the reference species on functional enrichment, sensitivity analyses were additionally performed using *Homo sapiens* and *Sus scrofa* as alternative reference organisms for GO and KEGG analyses. Analyses were performed independently for upregulated and downregulated genes. For GO analysis, the Biological Process (BP), Cellular Component (CC), and Molecular Function (MF) categories were evaluated, whereas enriched biological pathways were identified through KEGG analysis. *p*-values were adjusted using the Benjamini–Hochberg method and GO terms or KEGG pathways with an FDR < 0.05 were considered significantly enriched.

### 2.13. Protein–Protein Interaction (PPI) Network Analysis

Protein–protein interaction (PPI) networks were constructed in R using the STRINGdb package (v2.22.0) with the STRING database (v11.5) [40], using Bos taurus as the reference organism (taxid 9913) and a confidence score threshold of 0.4. Gene identifiers were mapped to the Bos taurus STRING database using the STRINGdb map function, and unmapped genes were excluded. The resulting networks were exported and visualized in Cytoscape (v3.10.4) [41], where functional modules were identified using the MCODE plugin (v2.0.3) with default parameters. Hub genes were subsequently identified using the cytoHubba plugin (v0.1) based on the maximal clique centrality (MCC) algorithm.

### 2.14. Statistical Analysis

Statistical analyses were performed in R. Ooplasm diameter was compared between the BCB+ and BCB− groups using a linear mixed-effects model (LMM) implemented with the lme4 package (v2.0.1), with BCB classification included as a fixed effect and collection date (six independent experiments) as a random effect. Differences in chromatin configuration between BCB+ and BCB− oocytes were evaluated using Fisher’s exact test based on oocytes analyzed in four independent experiments. Nuclear maturation rate was analyzed using a generalized linear mixed-effects model (GLMM) with a binomial distribution and logit link function, considering the experimental group as a fixed effect and collection date as a random effect. The significance of the group effect was assessed using a likelihood ratio test (LRT). When a significant group effect was detected, pairwise comparisons were performed using estimated marginal means (emmeans package, v2.0.3) with Tukey’s adjustment. For transcriptomic analyses, *p*-values obtained from differential gene expression and functional enrichment (GO and KEGG) analyses were adjusted using the Benjamini–Hochberg method to control the false discovery rate (FDR). Results with P or FDR < 0.05, as appropriate, were considered statistically significant.

## 3. Results

### 3.1. Characterization of BCB-Classified Oocytes

BCB staining, based on the activity of the enzyme glucose-6-phosphate dehydrogenase (G6PDH), allowed COCs to be classified as BCB+ or BCB− according to the retention or loss of cytoplasmic staining, respectively (Figure 1A). Of the 753 COCs recovered, 454 (60.3%) were classified as BCB+ and 299 (39.7%) as BCB−.

Ooplasm diameter was greater in BCB+ than in BCB− oocytes (131.1 ± 1.3 vs. 123.3 ± 1.2 µm; mean ± SEM; Figure 1B). The linear mixed-effects model estimated a mean difference of 7.86 µm between groups (95% CI: 4.56–11.15 µm; *p* < 0.001).

Among the 89 oocytes assessed for chromatin configuration, 23 (25.8%) showed no detectable DNA signal and were excluded from the analysis, corresponding to 15 of 52 BCB+ oocytes (28.8%) and 8 of 37 BCB− oocytes (21.6%). The exclusion rate did not differ significantly between groups (Fisher’s exact test, *p* = 0.473). The distribution of chromatin configurations differed between the BCB+ and BCB− groups (*p* < 0.001; Figure 1D). BCB− oocytes predominantly exhibited the CC2 configuration (58.6%), whereas BCB+ oocytes showed a higher proportion of the CC3 (43.2%) and CC4 (32.4%) configurations (Figure 1D).

### 3.2. Maturation of BCB-Classified Oocytes

Nuclear maturation was evaluated in a control group (incubation in maturation medium), a BCB control group (incubation in DPBS/PVA without BCB), and the BCB+ and BCB− groups (incubation in DPBS/PVA containing BCB). The generalized linear mixed-effects model revealed significant differences in nuclear maturation rate among the groups (likelihood ratio test, χ^2^ = 42.88, *p* < 0.001; Table 1). BCB− oocytes exhibited a lower maturation rate (7.4%) than the control (56.5%), BCB control (45.1%), and BCB+ (50.6%) groups (*p* < 0.05), whereas no differences were observed among the control, BCB control, and BCB+ groups (*p* > 0.05).

### 3.3. Data Quality of scRNA-Seq

To investigate the molecular basis underlying the ability of BCB staining to discriminate oocytes with different developmental competence, scRNA-seq was performed on four BCB+ and four BCB− immature oocytes. Sequencing generated an average of ~32 million read pairs per sample. After quality filtering, approximately 27 million read pairs were aligned to the reference genome, yielding 25,251,127 ± 4,028,655 uniquely mapped read pairs (89.4 ± 2.2%) (Table S1, mean ± SD).

### 3.4. Global Gene Expression Profile in BCB Oocytes

**Figure 2:**
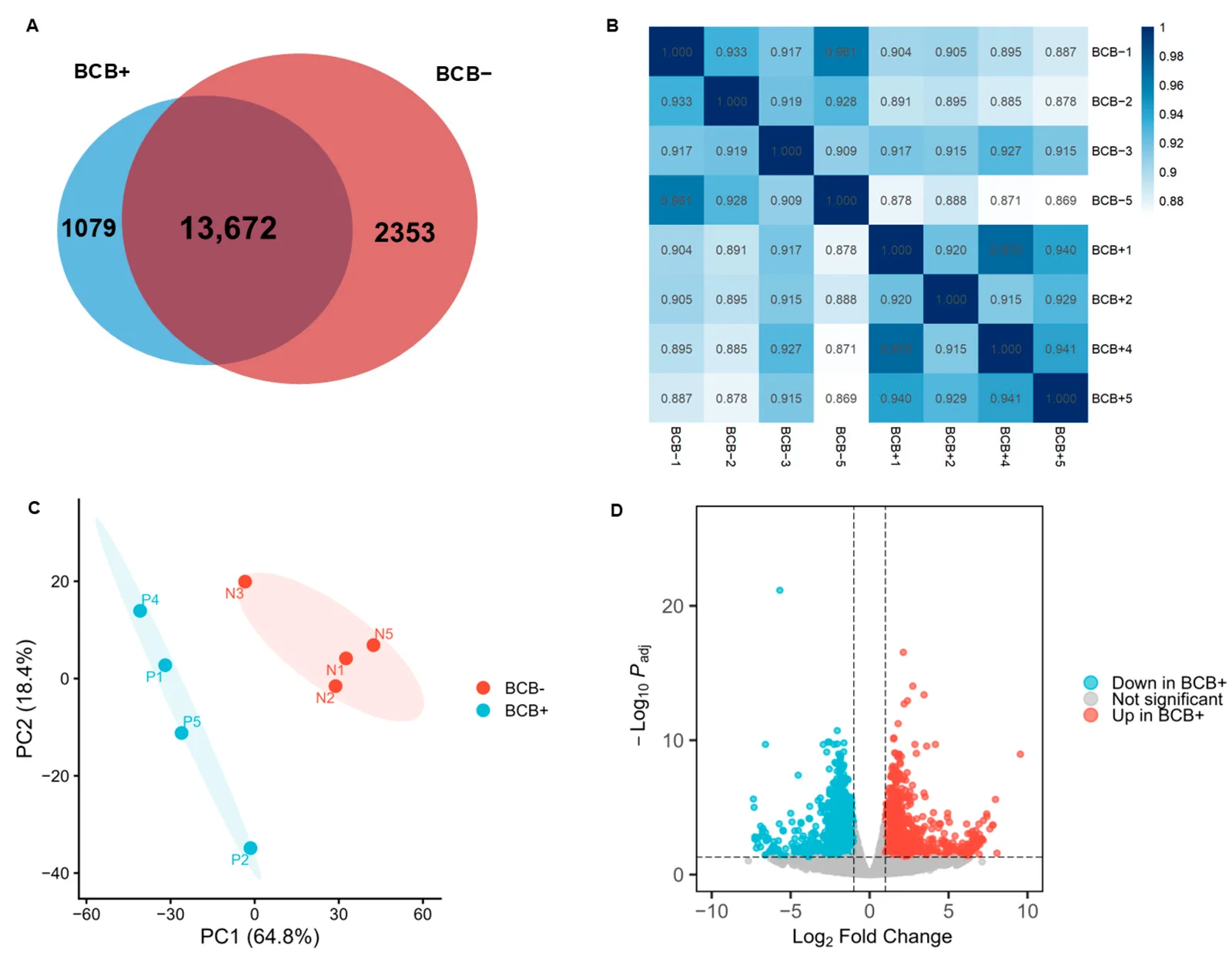
Transcriptomic profiles of BCB-classified alpaca oocytes. (**A**) Venn diagram showing the number of shared and uniquely expressed genes (FPKM > 1) in BCB+ and BCB− oocytes. (**B**) Pearson correlation matrix of gene expression profiles across all samples. (**C**) Principal component analysis (PCA) of BCB+ and BCB− oocytes. (**D**) Volcano plot showing differentially expressed genes (DEGs) in BCB+ compared to BCB− oocytes, identified using the thresholds of padj < 0.05 and |log_2_FC| > 1.

A total of 17,194 genes were detected (FPKM > 1) in at least one sample. Of these, 13,762 were detected in both groups, whereas 1079 and 2353 were detected only in BCB+ and BCB− oocytes, respectively (Figure 2A). The number of expressed genes per sample ranged from 11,941 to 12,568 in BCB+ oocytes and from 12,948 to 13,661 in BCB− oocytes. Pearson correlation analysis showed high similarity among the transcriptomic profiles of all samples (r = 0.869–0.970), with within-group correlations ranging from 0.915–0.970 in BCB+ and 0.909–0.961 in BCB− oocytes (Figure 2B). Principal component analysis (PCA) revealed a clear separation between the BCB+ and BCB− groups along principal component 1 (PC1), which explained 64.8% of the total variance, whereas principal component 2 (PC2) explained 18.4% (Figure 2C).

Analysis of the 10 most abundant transcripts revealed that seven transcripts were shared across all samples of both groups, including key regulators such as *PTTG1*, *CKS1B*, *MRPS14*, and *DYNLL1* (Table S2).

### 3.5. Differentially Expressed Genes

**Figure 3:**
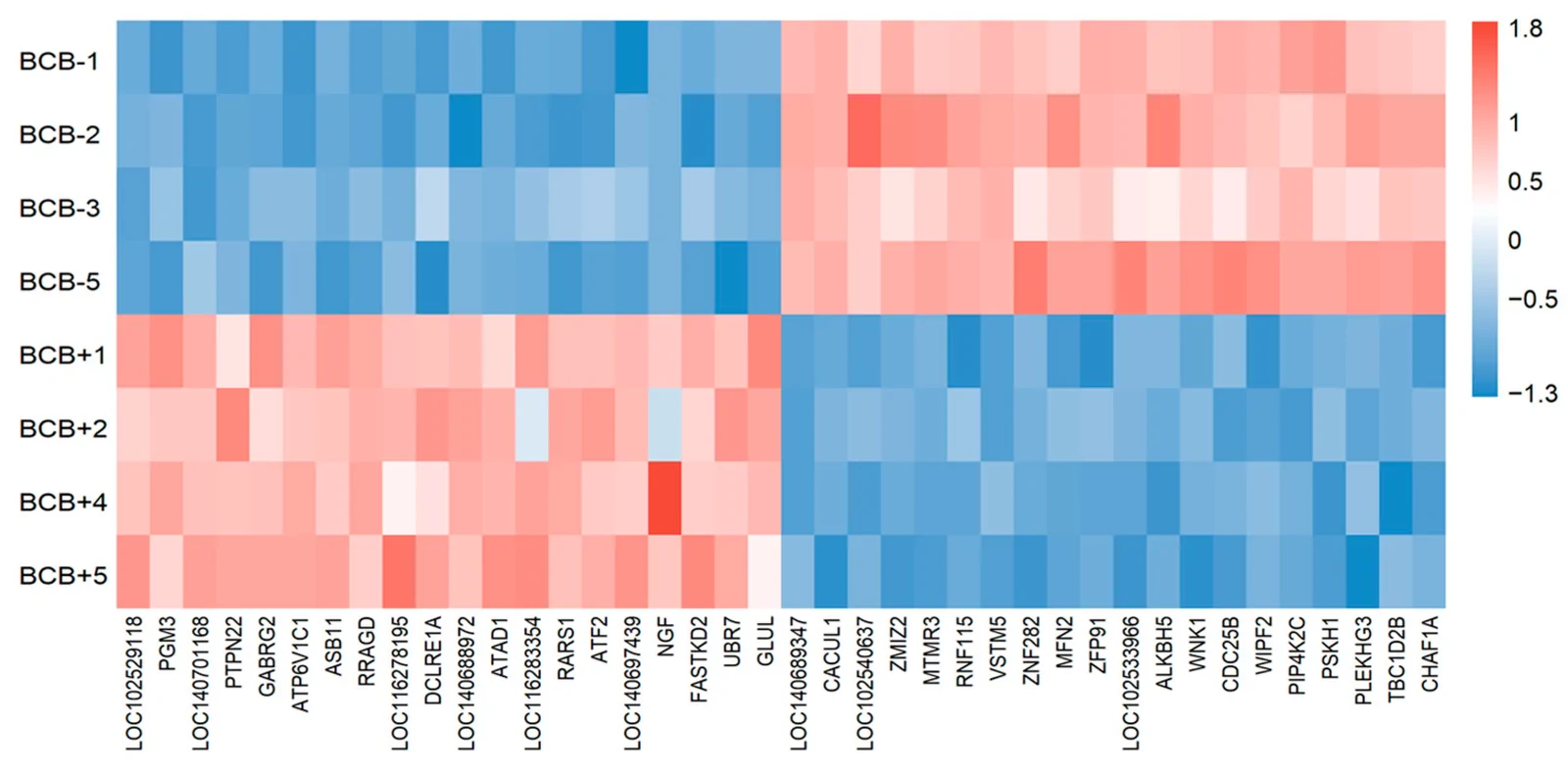
Heatmap of differentially expressed genes between BCB+ and BCB− alpaca oocytes. Heatmap showing the expression patterns of the most significant differentially expressed genes (DEGs), including the top genes upregulated and downregulated in BCB+ compared to BCB− oocytes, ranked by adjusted *p* value (padj). Gene expression values are presented as row Z-scores after scaling. Red indicates relatively higher expression, whereas blue indicates relatively lower expression across samples.

Differentially expressed genes (DEGs) between BCB+ and BCB− oocytes were identified using DESeq2. A total of 2978 DEGs were detected using the criteria of padj < 0.05 and |log_2_FC| > 1, of which 1453 were upregulated and 1525 were downregulated in BCB+ oocytes compared to BCB− oocytes (Figure 2D; Tables S3 and S4). The heatmap of the 40 most significant DEGs, corresponding to the 20 genes with the lowest padj values in each group, showed a clear separation between the two groups and included genes such as *FASTKD2* and *DCLRE1A* among those upregulated in BCB+ oocytes, and *ALKBH5* and *MFN2* among those downregulated (Figure 3).

To further assess the stability of the differential expression estimates, bootstrap sensitivity analysis showed a mean Spearman correlation of ρ = 0.855 across 100 iterations (median = 0.856; IQR = 0.819–0.894).

### 3.6. GO and KEGG Functional Enrichment Analysis

Mapping of alpaca gene symbols to bovine Entrez IDs resulted in 12,837 of 19,337 (66.4%) genes in the reference universe, 977 of 1453 (67.2%) genes upregulated in BCB+, and 1306 of 1525 (85.6%) genes downregulated in BCB+. Among the unmapped DEGs, 450/476 (94.5%) among upregulated genes and 209/219 (95.4%) among downregulated genes corresponded to LOC-designated loci. Non-coding RNAs represented 264/476 (55.5%) and 111/219 (50.7%) of the unmapped upregulated and downregulated genes, respectively, whereas protein-coding genes represented 170/476 (35.7%) and 89/219 (40.6%), respectively. Functional characterization of the unmapped protein-coding fraction using eggNOG-mapper recovered annotations associated with diverse cellular functions in both DEG sets (Table S5).

**Figure 4:**
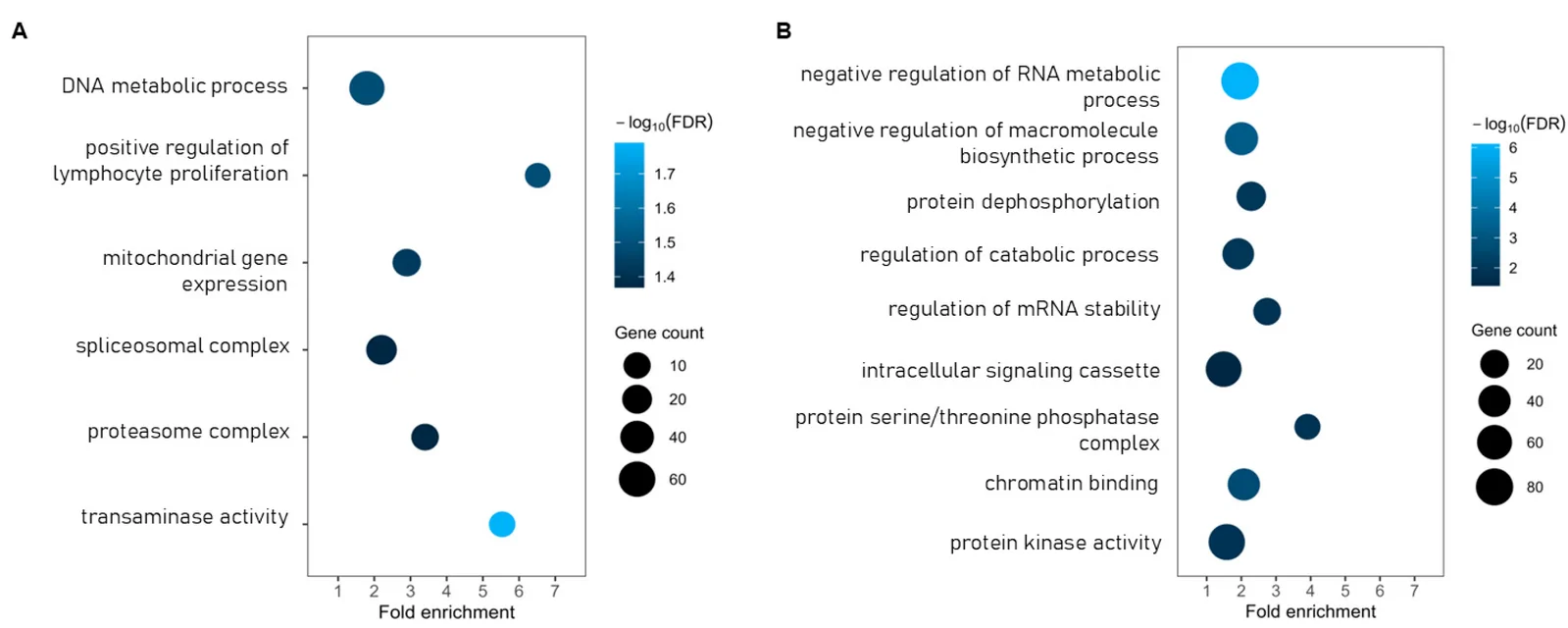
Gene Ontology (GO) functional enrichment analysis of differentially expressed genes. (**A**) Enriched Gene Ontology terms among genes upregulated in BCB+ oocytes. (**B**) Enriched Gene Ontology terms among genes downregulated in BCB+ oocytes. The x-axis represents fold enrichment, circle size indicates the number of genes associated with each GO term, and color represents the enrichment significance as −log_10_(FDR).

Functional enrichment analysis based on Gene Ontology (GO) was performed using the 2978 DEGs. Genes upregulated in BCB+ oocytes were significantly enriched in biological processes related to DNA metabolism, mitochondrial gene expression, and the positive regulation of cell proliferation. In the Cellular Component (CC) category, enriched terms were primarily associated with RNA processing (spliceosomal complex) and protein degradation (proteasome complex). In the Molecular Function (MF) category, the enriched terms were mainly related to the transfer of nitrogenous groups (transaminase activity and transferase activity, transferring nitrogenous groups) (Figure 4A; Table S6).

In contrast, genes downregulated in BCB+ oocytes were significantly enriched in biological processes associated with the regulation of macromolecule biosynthesis and RNA metabolism, regulation of catabolic processes, protein dephosphorylation, regulation of mRNA stability, and intracellular signaling pathways. In the CC category, enriched terms were predominantly associated with phosphatase complexes, whereas the MF category was enriched for functions related to chromatin binding and protein kinase activity (Figure 4B; Table S7).

Further KEGG enrichment analysis showed that genes upregulated in BCB+ oocytes were mainly involved in pathways related to the proteasome, cell cycle, and oocyte meiosis (Table S8). In contrast, genes downregulated in BCB+ oocytes were primarily associated with pathways involved in epigenetic regulation and intracellular signaling, including the Polycomb repressive complex, Hippo, Notch, ErbB, autophagy, and mRNA surveillance pathways (Table S9).

Cross-species sensitivity analyses using *Homo sapiens* and *Sus scrofa* as alternative reference organisms showed that several major functional patterns were conserved, although the number and identity of individual enriched GO terms and KEGG pathways varied among reference species (Table S10). Spliceosome-related functions were consistently enriched among genes upregulated in BCB+, whereas functions related to RNA regulation were consistently enriched among genes downregulated in BCB+. In the KEGG analysis, the cell cycle pathway was commonly enriched across all three reference organisms among genes upregulated in BCB+.

### 3.7. Protein–Protein Interaction (PPI) Analysis

**Figure 5:**
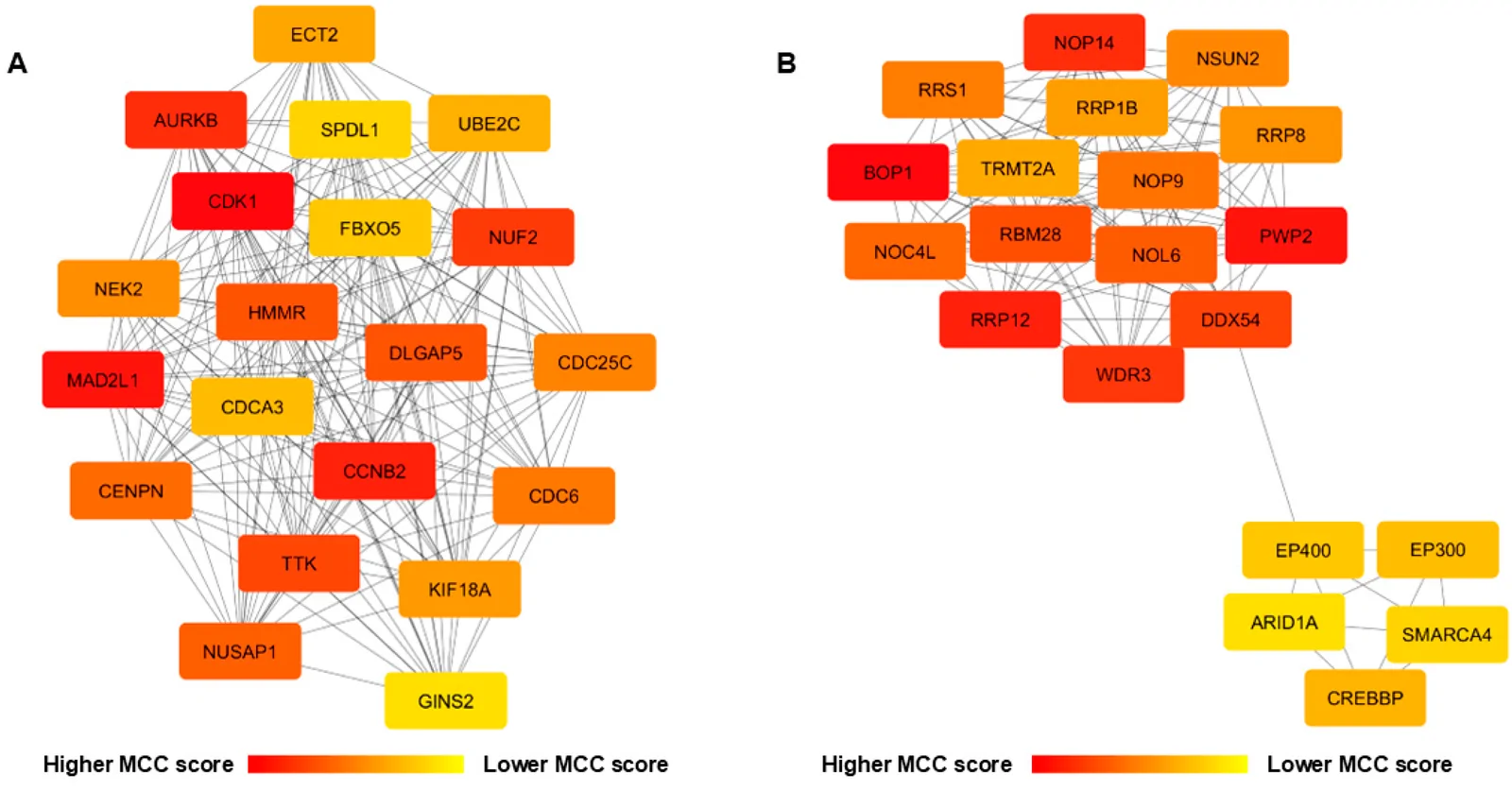
Protein–protein interaction (PPI) networks of the top 20 hub genes identified by cytoHubba using the Maximal Clique Centrality (MCC) algorithm. (**A**) Network of hub genes upregulated in BCB+ oocytes. (**B**) Network of hub genes downregulated in BCB+ oocytes. Node color represents the MCC score, with red indicating higher centrality and yellow indicating lower centrality.

Of the DEGs mapped to bovine Entrez IDs, 923/977 (94.5%) BCB+ genes and 1214/1306 (93.0%) BCB− genes were successfully mapped to the *Bos taurus* STRING database. Protein–protein interaction networks constructed from the DEGs comprised 886 nodes and 4339 interactions for genes upregulated in BCB+ oocytes, and 1268 nodes and 7559 interactions for genes downregulated in BCB+ oocytes. Centrality analysis using the MCC algorithm identified hub genes among those upregulated in BCB+ oocytes that were primarily associated with cell cycle progression and chromosome segregation, including *CDK1*, *CCNB2*, *MAD2L1*, and *AURKB*. In contrast, the top hub genes among those downregulated in BCB+ oocytes formed two distinct groups: one consisting of genes involved in ribosome biogenesis and RNA processing, including *NOP14*, *BOP1*, *RRP12*, and *NOP9*, and another comprising genes associated with chromatin remodeling and transcriptional regulation, including *EP300*, *CREBBP*, *SMARCA4*, *ARID1A*, and *EP400* (Figure 5).

## 4. Discussion

Identification of oocytes with high meiotic and developmental competence remains one of the major challenges in reproductive biology. In several species, Brilliant Cresyl Blue (BCB) staining, based on the activity of the enzyme glucose-6-phosphate dehydrogenase (G6PDH), has proven to be an effective tool for discriminating oocytes with different meiotic and developmental competence [15,17]. In alpacas, BCB staining has previously been associated with differences in maturation, cleavage, and blastocyst development rates [25]. However, the molecular basis underlying the differences between BCB+ and BCB− oocytes in this species has not yet been characterized. In the present study, we evaluated the morphometric characteristics and meiotic competence of alpaca BCB+ and BCB− oocytes and subsequently characterized their transcriptomic profiles using scRNA-seq.

First, we found that BCB-based selection allowed the identification of larger oocytes, with an ooplasm diameter of 131.1 ± 1.3 μm in BCB+ oocytes compared with 123.3 ± 1.2 μm in BCB− oocytes. Similar findings have been reported in other domestic species, including cattle [13], goats [42], pigs [43], and sheep [44], supporting the association between BCB-based selection and oocyte growth status. Oocyte growth and the progressive increase in oocyte diameter are closely associated with the acquisition of meiotic and developmental competence [45]. Consistent with this, BCB+ oocytes in our study exhibited a higher nuclear maturation rate than BCB− oocytes (50.6% vs. 7.4%). These results are consistent with our previous study in alpacas, in which BCB+ oocytes showed higher maturation than BCB− oocytes [25].

Although the MII rate observed in BCB− oocytes in the present study was particularly low (7.4%), considerable variability in the meiotic competence of BCB− oocytes has been reported among species and experimental conditions. MII rates of 0.8–3.6% and 13.7% have been reported in pigs [43,46], 37.0% in cats [18], 50.9% in sheep [44], 52.5% in goats [42], 59.2% in buffalo [47], and 65.7% in another porcine study [9], all lower than those observed in their corresponding BCB+ populations. In alpacas, our previous study also showed a low MII rate in BCB− oocytes (11.7%), although under different experimental conditions [25]. This variability may reflect biological differences among species and oocyte populations, as well as differences in experimental conditions and BCB classification criteria among studies.

Despite BCB exposure being proposed to influence oocyte physiology, its contribution to the reduced meiotic competence of BCB− oocytes remains unclear. Several studies have found no detrimental effect of BCB staining on nuclear maturation, whereas others have reported changes in oxidative status and mitochondrial parameters following exposure to the dye [48]. Importantly, these physiological changes do not appear to preferentially affect BCB− oocytes. Studies directly comparing BCB+ and BCB− populations have shown variable ROS responses, with higher levels reported in BCB− oocytes at specific experimental time points [43], but significantly higher levels in BCB+ oocytes in another study [49]. Moreover, Fonseka et al. [49] showed that modifying the cumulus-cell environment shifted a proportion of oocytes from the BCB− to the BCB+ phenotype and improved the maturation of BCB− oocytes from small follicles. Thus, although BCB exposure may induce physiological changes, the available evidence does not support a preferential deleterious effect of the dye on BCB− oocytes that would explain their particularly low maturation rate in the present study.

Likewise, the distribution of chromatin configurations (CC1–CC4) differed between the two groups. BCB+ oocytes predominantly exhibited chromatin partially or completely organized around a central nucleolus-like region (CC3, 43.2%) and a higher proportion of highly condensed chromatin (CC4, 32.4%) compared with BCB− oocytes (6.9% and 17.2%, respectively). In contrast, the CC2 configuration predominated in the BCB− group and was characterized by less condensed chromatin aggregates widely distributed throughout the nucleoplasm (58.6% vs. 21.6% in BCB+ oocytes). This pattern is consistent with that reported in mice [8] and pigs [50] where BCB+ oocytes more frequently exhibited chromatin configurations organized around the nucleolus (surrounded nucleolus, SN). In contrast, Murín et al. [43] did not observe differences in the proportion of oocytes with the SN configuration but did report a higher prevalence of diffuse chromatin in BCB− oocytes, like our findings. Together, these findings indicate that BCB-based selection is associated with differences in chromatin organization and condensation in GV oocytes. The transition from diffuse chromatin configurations to more condensed states is a characteristic event during oocyte growth and, in several mammalian species, has been associated with the progressive acquisition of meiotic and developmental competence, as well as with transcriptional silencing at the end of oocyte growth [51]. In this context, the predominance of more condensed chromatin configurations in BCB+ oocytes observed in our study is consistent with the higher nuclear maturation rate observed in this group.

These morphological and functional differences were accompanied by distinct transcriptomic features. The analysis of the global gene expression profile revealed a set of highly abundant transcripts shared between BCB+ and BCB− oocytes, among which *PTTG1*, *CKS1B*, *MRPS14*, *GSTM3*, and *DYNLL1* were the most prominent. High expression of these genes has also been reported in oocytes from other mammalian species, including cattle (*PTTG1*, *GSTM3*) [52] and pigs (*DYNLL1*) [21], suggesting the existence of a conserved transcriptomic program during the germinal vesicle stage. Collectively, these genes are involved in fundamental processes essential for oocyte physiology, including cell cycle regulation (*PTTG1* and *CKS1B*) [53,54,55,56], translation and mitochondrial function (*MRPS14*) [57,58], as well as cytoskeletal organization and meiotic spindle assembly (*DYNLL1*) [59]. The high expression of these transcripts in both groups suggests that these processes represent core functions shared by germinal vesicle-stage oocytes, regardless of their classification by BCB staining.

In contrast to this shared basal transcriptomic program, transcriptomic analysis revealed differences in gene expression between BCB+ and BCB− oocytes, as reflected by the clear separation of the two groups in the principal component analysis and the identification of differentially expressed genes. These differences were accompanied by distinct functional profiles. Functional enrichment analysis of the genes with higher expression in BCB+ oocytes revealed enrichment of processes related to DNA metabolism, cell proliferation, and mitochondrial gene expression, as well as components of the proteasome and spliceosomal complex. In addition, KEGG analysis identified enrichment of the cell cycle, oocyte meiosis, and proteasome pathways. These functional differences were also reflected in the PPI network, where *CDK1*, *CCNB2*, *MAD2L1* and *AURKB* were identified among the main hub genes, showing convergence toward processes related to cell cycle regulation, meiotic progression, and chromosomal dynamics.

Previous studies using BCB to compare the molecular profiles of immature oocytes have reported findings that are partially consistent with our results. In cattle, Torner et al. [22] found that BCB+ oocytes exhibited a higher abundance of transcripts related to the cell cycle, transcriptional regulation, and protein biosynthesis. Likewise, in pigs, Liu et al. [21] identified 155 DEGs between GV-stage BCB+ and BCB− oocytes, which were enriched in pathways related to cell cycle regulation, oocyte meiosis, spliceosome assembly, and nucleotide excision repair. In addition, a higher abundance of genes associated with mitochondrial biogenesis and function has also been reported in immature bovine BCB+ oocytes compared with BCB− oocytes [17]. Among the hub genes identified in the present study, *CDK1* and *CCNB2* deserve particular attention because they are components of the Cyclin B/CDK1 regulatory axis, a key regulator of meiotic progression. Functional studies in mouse oocytes have shown that *CCNB2* contributes to MPF/CDK1 activation during meiotic resumption [60], whereas its depletion disrupts spindle assembly, chromosome segregation, and normal meiotic progression [61]. Consistent with the importance of this regulatory module, *CCNB2* has also been associated with embryonic competence in cattle [57]. Similarly, in camelids, BCB+ oocytes exhibit higher expression of *CDK1* together with *CCNB1*, another member of the Cyclin B family, compared with BCB− oocytes [15].

Similar transcriptomic features have also been described in other models of oocyte competence acquisition. In bovine GV oocytes, genes associated with cell cycle regulation were enriched in oocytes with greater developmental competence recovered from medium follicles compared with those from small follicles [62]. Likewise, Latorraca et al. [52] reported that larger growing oocytes exhibit progressive enrichment of transcriptomic programs associated with chromosome segregation, DNA repair, and meiotic cell cycle progression.

However, transcriptomic differences between these two populations have not been consistently observed. Walker et al. [10] did not detect differentially expressed genes between bovine BCB+ and BCB− oocytes in a context where the difference in blastocyst development between the two groups, although significant, was relatively modest (18% vs. 9%). This contrasts with the marked transcriptomic separation observed in our study, in which 2978 differentially expressed genes (DEGs) were identified between BCB+ and BCB− oocytes. This discrepancy may be attributable to multiple factors, including differences among species, experimental designs, and oocyte selection criteria. In our study, selection was restricted to oocytes with clearly distinguishable BCB phenotypes, excluding intermediate staining patterns, and this separation was accompanied by a marked difference in the ability to reach the MII stage (50.6% vs. 7.4%). Taken together, methodological and biological differences may have contributed to increasing the biological contrast between the two groups and, consequently, facilitated the detection of transcriptomic differences, although this interpretation should be made with caution.

During oocyte growth, the acquisition of developmental competence is accompanied by extensive transcriptomic reprogramming. As oocytes complete their growth phase, transcriptional activity progressively declines, chromatin becomes increasingly condensed, and maternal transcripts required to sustain meiotic maturation and early embryonic development accumulate [4]. In this context, it is noteworthy that BCB− oocytes exhibited a broader transcriptomic profile, characterized by a higher number of expressed genes, a greater number of exclusively expressed genes, and enrichment of a wider range of biological processes and signaling pathways compared with BCB+ oocytes. Specifically, genes with higher expression in BCB− oocytes were enriched in processes related to the negative regulation of gene expression and macromolecule biosynthesis, RNA metabolism, mRNA stability, protein dephosphorylation, and chromatin binding. Consistent with these findings, KEGG analysis revealed enrichment of pathways involved in epigenetic regulation, RNA surveillance, and intracellular signaling, including the Polycomb repressive complex, Hippo, Notch, and ErbB pathways. PPI network analysis further supported these findings by identifying two major functional modules, comprising genes involved in ribosome biogenesis and RNA processing, as well as chromatin remodeling and transcriptional regulation, including *EP300*, *CREBBP*, *SMARCA4*, and *ARID1A*. The interpretation of these findings is particularly relevant when considering the dynamic nature of the transcriptomic changes that occur during oocyte growth. In this regard, Latorraca et al. [52] demonstrated that the oocyte transcriptome undergoes progressive reorganization throughout oocyte growth, culminating in a marked transcriptomic transition between oocytes measuring 100–109 μm and 110–119 μm in diameter. This transition is characterized by the replacement of programs primarily associated with cytoplasmic activities, such as RNA processing and metabolism, ribosome biogenesis, and intracellular transport, by predominantly nuclear programs related to chromosome segregation, DNA repair, and the meiotic cell cycle. In this context, the greater representation of processes related to RNA metabolism, ribosome biogenesis, and regulation of gene expression observed in BCB− oocytes may be consistent with a transcriptomic state corresponding to a less advanced stage of oocyte growth. Nevertheless, it is also important to consider that the higher relative abundance of these genes in BCB− oocytes may simply reflect a lower abundance of these same genes and their associated biological processes in BCB+ oocytes, which exhibited a higher maturation rate, consistent with the transcriptional silencing that characterizes the final stages of oocyte growth.

Interestingly, although BCB classification is based on G6PDH activity, *G6PD* was not differentially expressed between BCB+ and BCB− oocytes in the present study. Similarly, other major PPP-related genes, including *PGLS*, *PGD*, *TKT*, *TALDO1*, and *RPIA*, did not show differential expression. Previous transcriptomic studies of BCB-classified oocytes have likewise not identified *G6PD* expression or the PPP among the major transcriptional differences associated with the BCB phenotype [10,21]. This finding highlights that BCB staining reflects G6PDH enzymatic activity rather than *G6PD* transcript abundance; therefore, differences in the BCB phenotype do not necessarily imply differences in *G6PD* mRNA levels. The differences in G6PDH activity underlying BCB classification may involve regulatory mechanisms not captured by transcript abundance alone. Notably, recent studies in porcine oocyte–cumulus complexes have reported differences in the expression of PPP-related genes in cumulus cells [63], while pre-culture of BCB− oocytes with healthy cumulus cell masses induced a shift toward the BCB+ phenotype and improved their meiotic competence [49]. Together, these observations suggest that understanding the molecular basis of the BCB phenotype may require considering not only the oocyte transcriptome but also its metabolic interactions with the surrounding cumulus cells.

In addition to these biological considerations, the potential influence of ex vivo ovarian transport conditions should be considered. Prolonged ovarian storage can also affect oocyte quality and meiotic competence, as well as cellular and molecular characteristics such as chromatin configuration, oxidative status, mitochondrial function, and gene expression [64,65,66,67]. However, all BCB+ and BCB− oocytes were obtained from ovaries subjected to the same transport conditions before BCB classification. Thus, although transport may have influenced the overall physiological and molecular state of the recovered oocytes, its specific contribution to the differences observed between BCB+ and BCB− oocytes cannot be determined, nor can we exclude the possibility that these two populations responded differently to these conditions. Likewise, the potential effects specifically attributable to the change in altitude during transport were not independently assessed. Nevertheless, these conditions form part of the context in which reproductive research in alpacas is conducted, a species native to high-altitude Andean regions in which oocyte competence and the efficiency of in vitro embryo production remain important limitations for the development of ARTs [68].

Another methodological consideration is the use of *Bos taurus* as the reference organism for functional enrichment analyses because of the limited functional annotation available for *Vicugna pacos*. Cross-species sensitivity analyses using human and pig annotations supported several of the main functional patterns identified with the bovine reference, particularly spliceosome-related functions and cell-cycle-associated pathways among genes upregulated in BCB+, and RNA regulatory processes among genes downregulated in BCB+. However, differences in the number and identity of enriched terms and pathways were observed among reference species, indicating that some functional interpretations are dependent on the annotation background used.

The sample size used for single-oocyte RNA-seq (*n* = 4 per group) should also be considered when interpreting the transcriptomic results, as it reduces statistical power, particularly for detecting genes with small or moderate expression differences. Methodological benchmarking indicates that reduced biological replication primarily decreases sensitivity and the ability to detect true differential expression, whereas false-positive rates remain relatively well controlled by methods such as DESeq2 [69,70]. Furthermore, in scRNA-seq data, DESeq2 has been shown to provide better control of false positives when the biological replicate structure is preserved [71]. This is particularly relevant for single-oocyte transcriptomics, as mammalian oocytes contain substantially higher amounts of endogenous RNA than conventional single cells, and single-oocyte RNA-seq has shown high reproducibility for transcriptome characterization [72]. In the present study, bootstrap sensitivity analysis showed moderate stability of the overall differential expression estimates (mean Spearman ρ = 0.855; median = 0.856; IQR = 0.819–0.894 across 100 iterations). According to Degen and Medo [37], the mean correlation remained above the range associated with greater sensitivity to changes in cohort composition (ρ < 0.8). Given that functional enrichment results may be more stable than individual gene-level results in small cohorts [37], the broader biological patterns identified in our study may be less sensitive to the limited sample size than individual DEG estimates.

Moreover, the transcriptomic findings were consistent with independently obtained phenotypic differences between BCB+ and BCB− oocytes, including oocyte diameter, chromatin configuration, and the ability to reach MII. This consistency supports the biological relevance of the broader functional patterns identified in the transcriptomic analysis. However, the transcriptomic findings were not independently validated by RT-qPCR or protein-based approaches. Therefore, individual DEGs and hub genes should be interpreted as candidates, and their differential expression and functional relevance require confirmation in larger, independent biological samples.

From an applied perspective, the molecular differences identified between BCB+ and BCB− oocytes provide a basis for investigating strategies to improve alpaca IVM systems. In particular, candidate genes and pathways associated with cell-cycle regulation, mitochondrial function, and RNA processing represent potential molecular targets for exploring whether modulation of these processes can improve oocyte maturation. Functional studies of these candidates could further determine their contribution to meiotic competence and subsequent embryo development, ultimately contributing to the refinement of in vitro embryo production protocols in alpacas.

## 5. Conclusions

This study provides new insights into the transcriptomic changes associated with meiotic competence in alpaca oocytes selected by BCB staining. Our findings reveal coordinated differences in key processes, including the cell cycle, meiotic regulation, mitochondrial function, RNA processing, and gene expression control. Furthermore, observed differences in ooplasm diameter, chromatin organization, and meiotic competence support the biological relevance of these transcriptomic profiles. This is the first study to characterize the transcriptome of BCB-selected alpaca oocytes at single-cell resolution, establishing a foundation for future research aimed at elucidating the mechanisms regulating oocyte competence and optimizing in vitro embryo production systems in this species.

## Figures and Tables

**Table 1:** Nuclear maturation rate of BCB-classified alpaca oocytes.

Group	No. of Oocytes	Matured Oocytes (%)
Control	109	56.5 ± 6.5 ^a^
BCB control	60	45.1 ± 4.0 ^a^
BCB+	61	50.6 ± 8.1 ^a^
BCB−	56	7.4 ± 3.2 ^b^

Values are presented as mean ± SEM from at least 5 independent biological replicates. Different superscript letters indicate significant differences among groups (*p* < 0.05).

## Data Availability

The sequencing data generated in this study have been deposited in the Gene Expression Omnibus (GEO) under accession number GSE348962.

## Supplementary Materials

The following supporting information can be downloaded at https://www.mdpi.com/article/10.3390/ani16193081/s1, Table S1: Summary of single-cell RNA-seq reads after quality filtering and alignment to the alpaca reference genome; Table S2: Top 10 most abundantly expressed genes in BCB− and BCB+ oocytes; Table S3: Differentially expressed genes upregulated in BCB+ oocytes; Table S4: Differentially expressed genes downregulated in BCB+ oocytes; Table S5: Orthology-based functional annotation of unmapped genes using eggNOG-mapper; Table S6: Gene Ontology (GO) enrichment analysis of upregulated genes in BCB+ oocytes; Table S7: Gene Ontology (GO) enrichment analysis of downregulated genes in BCB+ oocytes; Table S8: KEGG pathway enrichment analysis of genes upregulated in BCB+ oocytes; Table S9: KEGG pathway enrichment analysis of genes downregulated in BCB+ oocytes; Table S10: Cross-species sensitivity analysis of Gene Ontology (GO) enrichment using *Homo sapiens* and *Sus scrofa*.
